# Supplementary figures and images for: Interplay between chemotaxis, quorum sensing, and metabolism regulates Escherichia coli-Salmonella Typhimurium interactions in vivo
Source: PLoS Pathog. 2025 May 2;21(5):e1013156. doi: 10.1371/journal.ppat.1013156 (PMC12074654; doi:10.1371/journal.ppat.1013156)

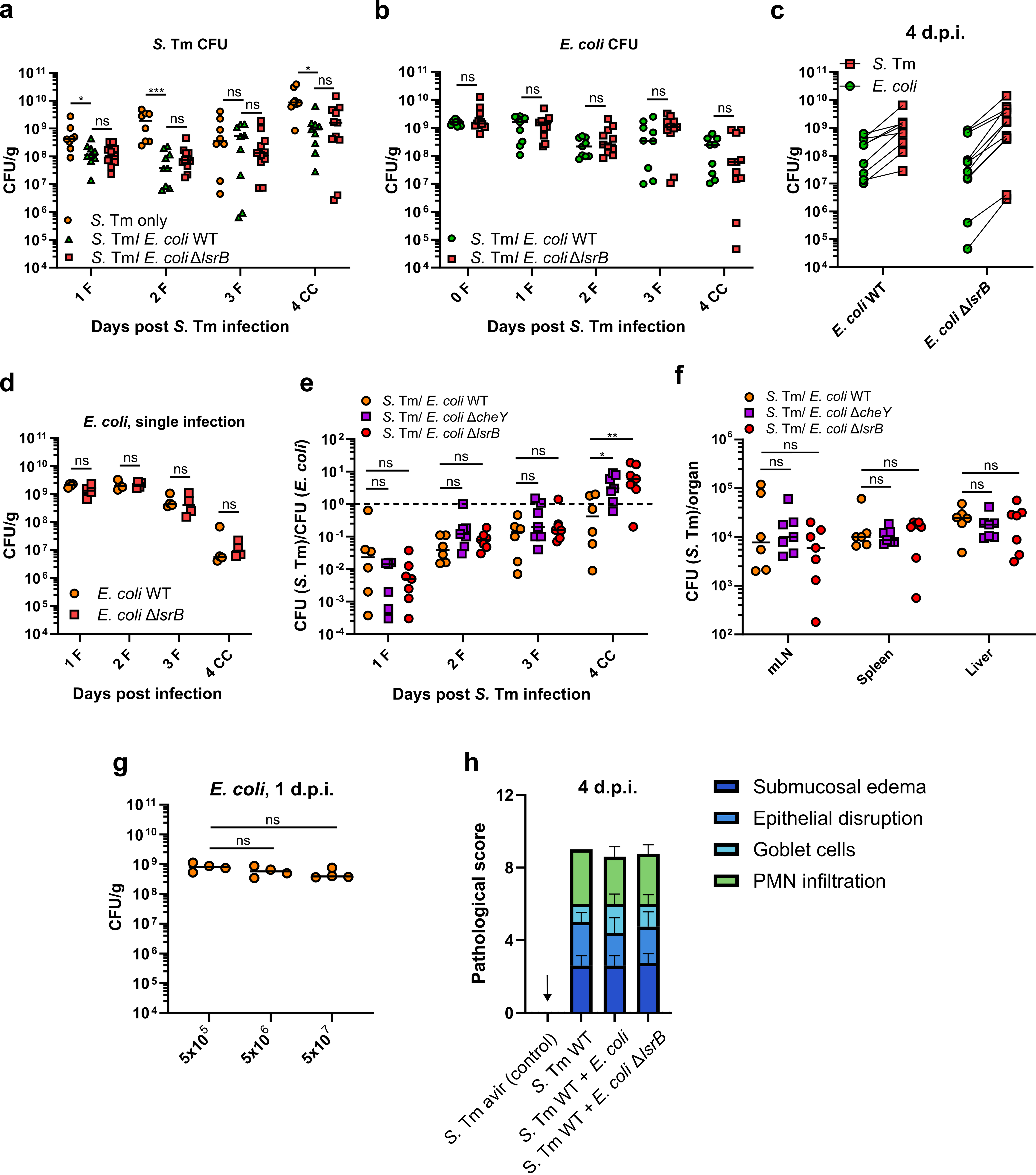

Supplement: S1 Fig — (a) Colony forming units (CFU) per gram of feces counts of S. Tm in feces (F) and cecal content (CC) of mice infected with either S. Tm only or precolonized with E. coli wild type or ΔlsrB strains, as seen in Fig 1b. The lines indicate median values (min mice n = 8, at least two independent experiments). P values were calculated using the Kruskal-Wallis test with post hoc correction for false discovery rate (adjusted *** ≙ P < 0.0005, * ≙ P < 0.05, ns – not significant). (b) CFU per gram of feces counts of E. coli wild type or ΔlsrB strains in feces (F) and cecal content (CC) of mice infected with S.Tm, as seen in Fig 1b. The lines indicate median values (min mice n = 9, at least two independent experiments). P values were calculated using the two-tailed Mann-Whitney U-test (ns – not significant). (c) CFU per gram of feces counts of E. coli and S. Tm in mice precolonized with E. coli Z1331 wild-type or ΔlsrB at 4 days post S. Tm infection, as seen in Fig 1b. (d) Colonization dynamics of streptomycin-pretreated mice by E. coli Z1331 wild-type and ΔlsrB strains in single infections, measured as CFU/g in feces (F) and cecal content (CC). The lines indicate median values (mice n = 4 in one experiment). P values were calculated using the two-tailed Mann-Whitney U-test (ns – not significant). (e) Competitive infections of S. Tm SL1344 against resident E. coli Z1331 wild-type, chemotaxis-deficient ΔcheY or AI-2 chemotaxis-negative ΔlsrB mutant strain in ampicillin-pretreated C57BL/6J SPF mice. The lines indicate median values (min mice n = 6, at least two independent experiments). P values were calculated using the Kruskal-Wallis test with post hoc correction for false discovery rate (adjusted ** ≙ P < 0.005, * ≙ P < 0.05, ns – not significant). The dashed line indicates the competitive index (CI) value of 1. F, feces; CC, cecal content. (f) S. Tm counts in mesenteric lymph nodes (mLN), spleen and liver of S. Tm-infected mice as seen in panel (e). The lines indicate median [file ppat.1013156.s001.tif]

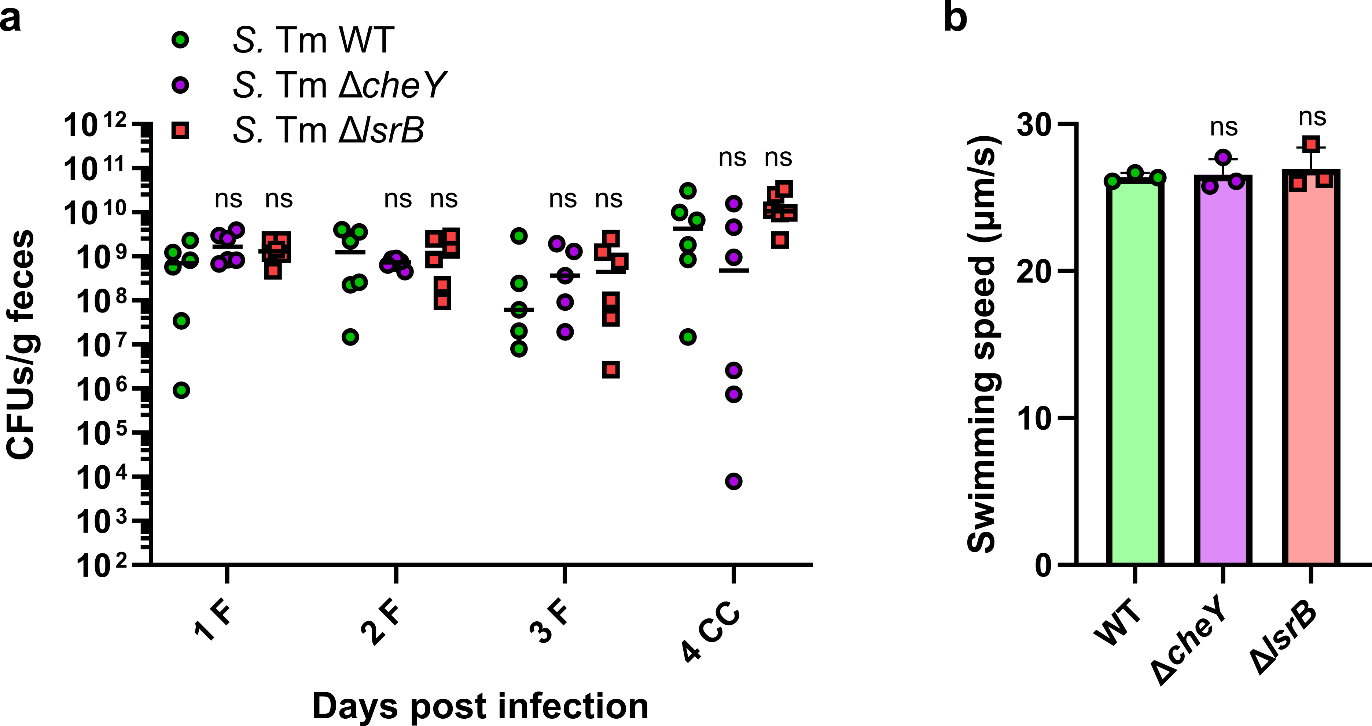

Supplement: S2 Fig — (a) Colonization dynamics of streptomycin-pretreated mice by S. Tm SL1344 wild-type (WT), ΔcheY and ΔlsrB strains in single infections, measured as CFU/g in feces (F) and cecal content (CC). The lines indicate median values (min mice n = 5 in one experiment). P values were calculated using the Kruskal-Wallis test with post hoc correction for false discovery rate (ns – not significant). (b) Swimming speed measurements of S. Tm wild-type (WT), ΔcheY and ΔlsrB strains grown in TB medium (n = 3, three independent experiments), analyzed with the tracking algorithm (see Materials and Methods). P values were calculated using the unpaired t-test (ns – not significant). (TIF) [file ppat.1013156.s002.tif]

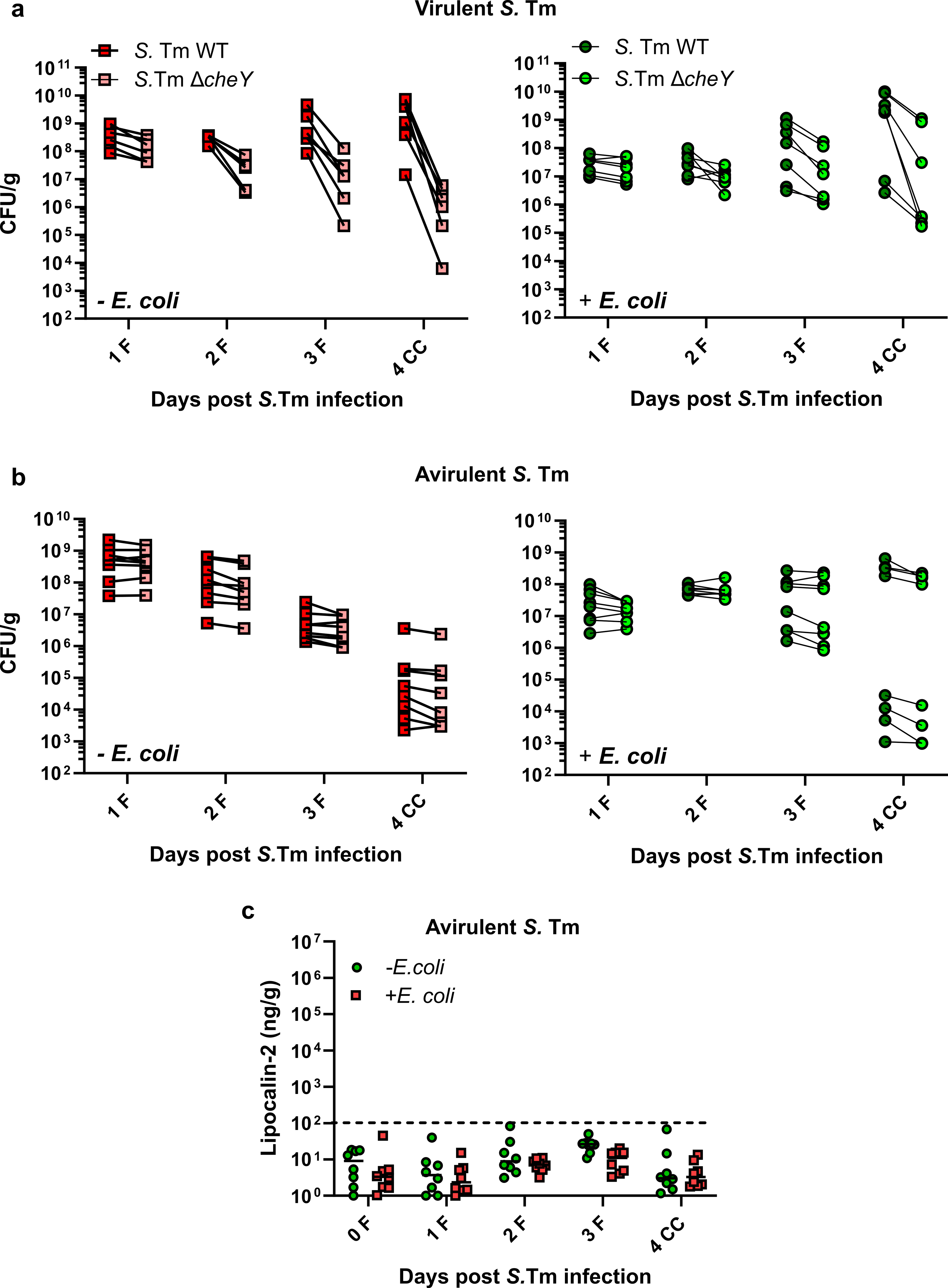

Supplement: S3 Fig — Colony forming units (CFU) counts of S. Tm SL1344 wild-type and chemotaxis-deficient ΔcheY strains in (a) virulent and (b) avirulent ΔinvG ΔsseD background. Mice were either infected with S. Tm only or were precolonized with E. coli according to the experimental scheme shown in Fig 1a. The gradual loss of CFU counts in avirulent S. Tm is due to its compromised ability to compete against the regrowing microbiota. (c) Lipocalin-2 levels per gram of feces (F) and cecal content (CC) of mice infected with avirulent S. Tm SL1344 ΔinvG ΔsseD. Dashed line indicates approximate level of lipocalin-2 marking a shift towards gut inflammation. Lines indicate median values (mice n = 8, at least two independent experiments). (TIF) [file ppat.1013156.s003.tif]

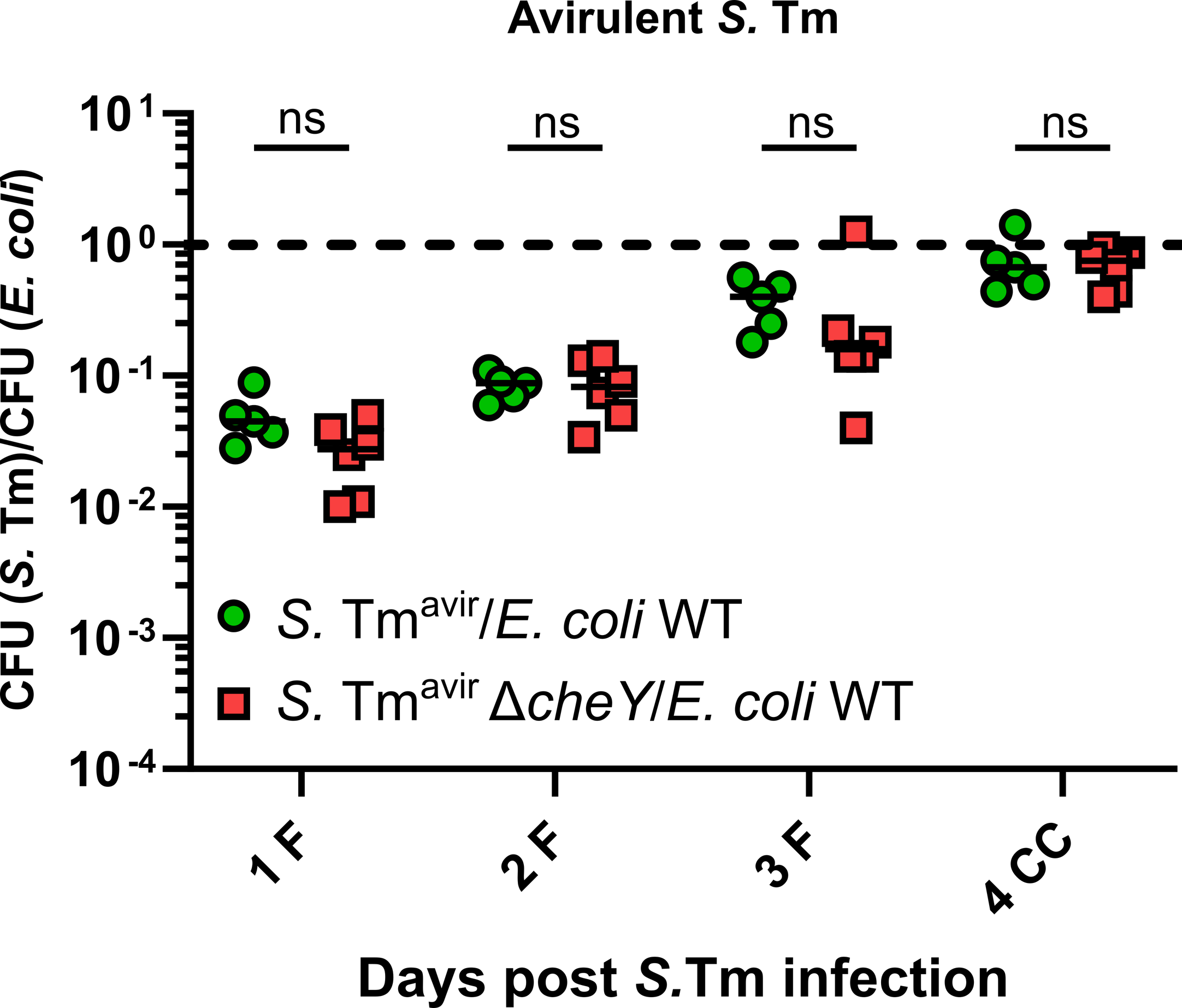

Supplement: S4 Fig — Competitive infection of avirulent S. Tm SL1344 ΔinvG ΔsseD strain (WT) and its non-chemotactic ΔcheY knockout strain against resident E. coli Z1331 strain. The lines indicate median values (min mice n = 5, at least two independent experiments). P values were calculated using the two-tailed Mann-Whitney U-test (ns – not significant). The dashed line indicates the competitive index value of 1. F, feces; CC, cecal content. (TIF) [file ppat.1013156.s004.tif]

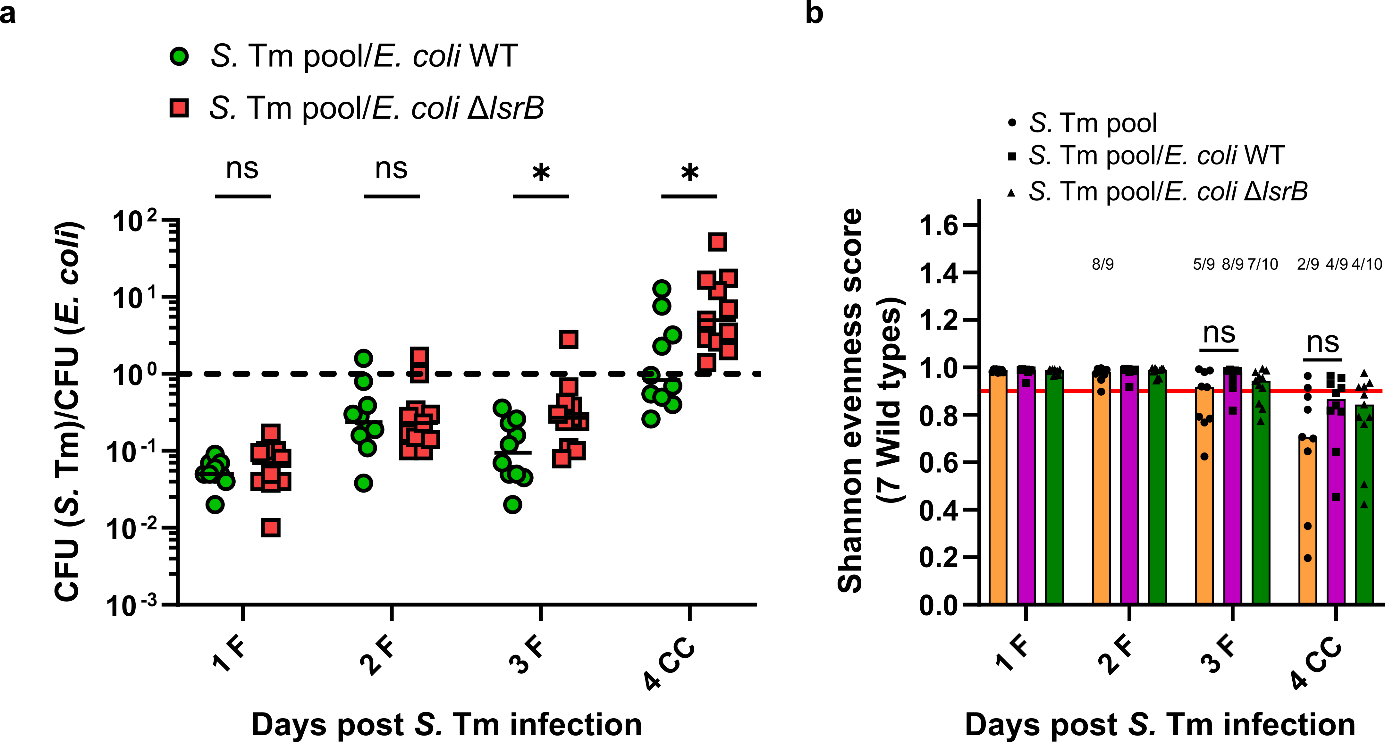

Supplement: S5 Fig — (a) Competitive infections of S. Tm SL1344 WISH-tagged strain pool against resident E. coli Z1331 wild-type or AI-2 chemotaxis-negative ΔlsrB mutant strain. The lines indicate median values (min mice n = 10, at least two independent experiments). P values were calculated using the two-tailed Mann-Whitney U-test (* ≙ P < 0.05, ns – not significant). The dashed line indicates the competitive index value of 1. F, feces; CC, cecal content. (b) Shannon evenness score (SES) was calculated for the 7 WISH-barcoded SL1344 wild types. The red line indicates the SES of 0.9, which was the cutoff for further analysis. The number above the bar indicates how many samples are within this threshold. (TIF) [file ppat.1013156.s005.tif]

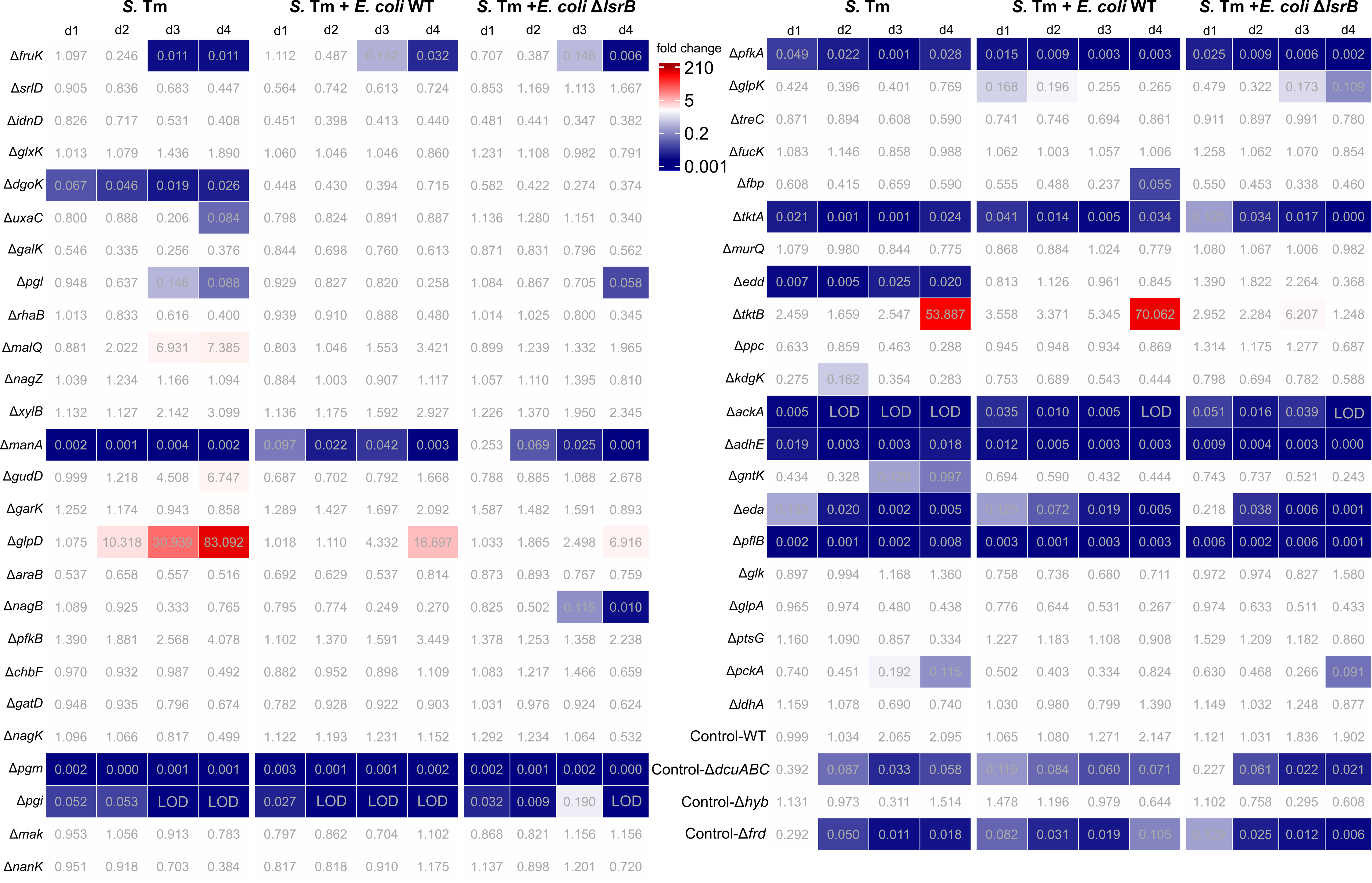

Supplement: S6 Fig — A heatmap showing the fitness of each S. Tm mutant in single infections and in competition with indicated E. coli strains. The shades of blue indicate loss of fitness, whereas the shades of red indicate gain of fitness, and white indicates a neutral effect. The competitive index values of all metabolic mutants tested are listed in Table S1. LOD, limit of detection as described in Materials and Methods. (TIF) [file ppat.1013156.s006.tif]

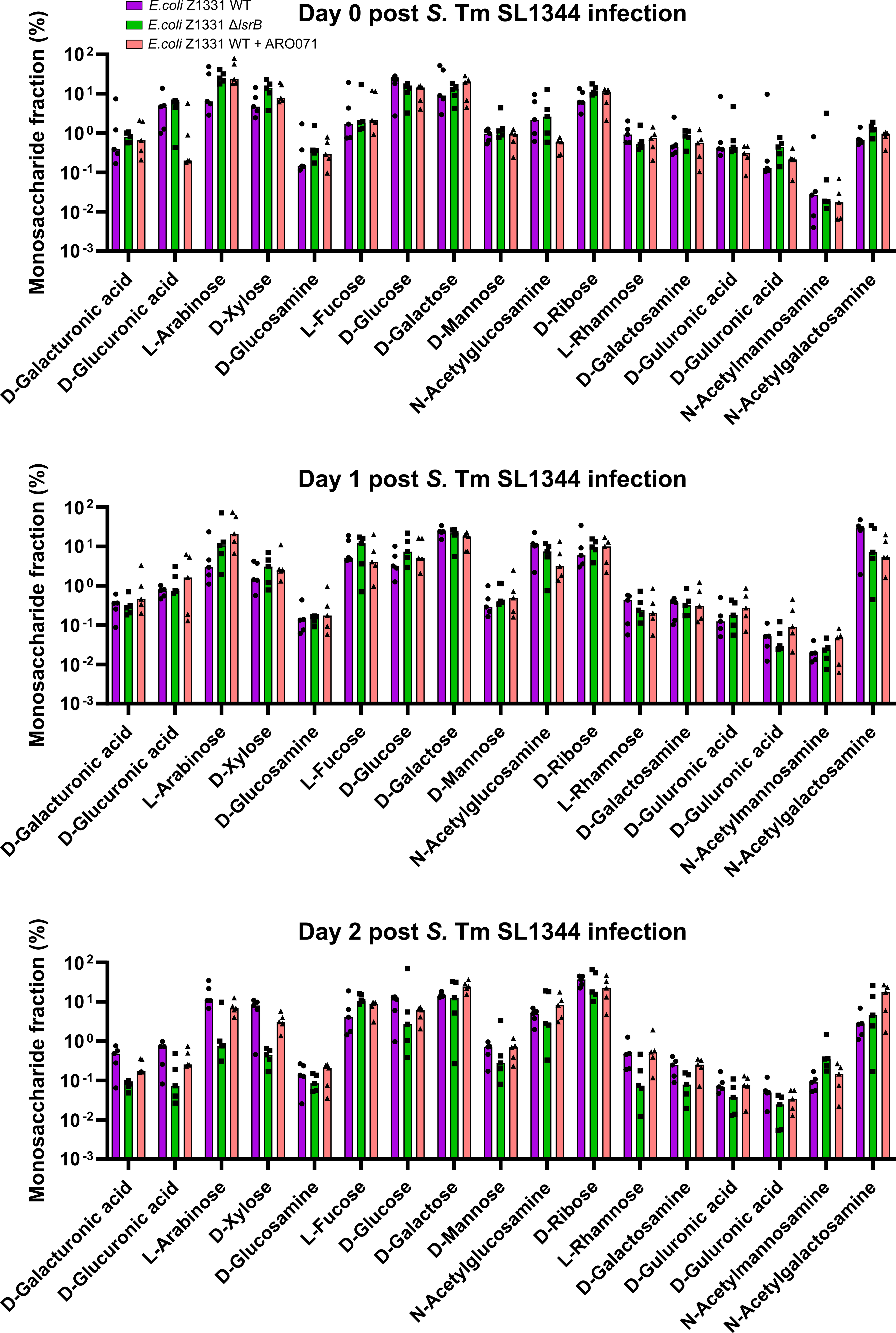

Supplement: S7 Fig — Streptomycin-pretreated SPF C57BL/6J mice were precolonized with either E. coli Z1331 wild-type, E. coli Z1331 ΔlsrB, or a combination of E. coli Z1331 wild-type and E. coli ARO071 (an AI-2 overproducing strain). Following precolonization, all groups were infected with wild-type S. Tm. The sample size per group was n = 5, derived from two independent experiments. For comparison, a control group (day 0) was precolonized with E. coli but was not inoculated with S. Tm. The groups are indicated above the respective plot. Cecal content was analyzed using LC-MS (see Methods). Data are presented as bar plots, displaying the median value along with individual data points. Monosaccharides are plotted as a fraction of the total monosaccharide content in each sample, expressed as a percentage, calculated using the formula: (monosaccharide/Σ(all monosaccharides)) x 100. (TIF) [file ppat.1013156.s007.tif]
