# Supplementary material for: Interplay between chemotaxis, quorum sensing, and metabolism regulates Escherichia coli-Salmonella Typhimurium interactions in vivo
Source: PLoS Pathog. 2025 May 2;21(5):e1013156. doi: 10.1371/journal.ppat.1013156 (PMC12074654; doi:10.1371/journal.ppat.1013156)
Supplement: Attachment — Submitted filename: Response to the reviewers.docx [file ppat.1013156.s016.docx]

**Reviewer 1**

In this manuscript, Laganenka et al. report that sensing of and chemotaxis towards AI-2 allows E. coli to compete with S. Typhimurium (S. Tm) for limiting nutrients. In antibiotic (streptomycin or ampicillin)-treated mice, precolonization with the E. coli wild-type reduces intestinal colonization with S. Tm at various time points after infection, while a mutant unable to sense AI-2 confers less protection. Consistent with previous reports, the presence of E. coli reduced S. Tm induced inflammatory responses. Furthermore, chemotaxis was required for S. Tm to efficiently compete against E. coli, in line with previous reports from the Hardt group, however, AI-2 sensing in S. Tm was dispensable. They next hypothesize, based on previous data that AI-2 sensing allows E. coli strains to inhabit different niches in the gut, that the E. coli wild-type and the lsrB mutant might differentially affect S. Tm metabolism as they are competing for different (metabolic?) niches. Using a curated library of mutants, the authors show that this is not the case, and only marginal effects are observed for S. Tm mutants deficient in mannose utilization and C4-dicarboxylate antiporters.
The question as to how the gut microbiota contributes to colonization resistance against enteric pathogens is very timely and of interest to a wide audience, and as such, this study attempts to address an interesting question. The writing is clear and concise.
My main concern is that the study is somewhat preliminary, and substantial experimentation would be required to support major conclusions.

Author response: We thank the reviewer for finding our work interesting and timely, and for the helpful suggestions for improving the manuscript.

**Major points:**

1. **The first part of the manuscript, Fig. 1-3, is somewhat predictable based on previous work by the Hardt lab and others. While the exact experiments are not the same, the outcome is very predictable and does not reveal much new information. The more exciting aspect of this work is the mutant screen, which has the potential to uncover some interesting biology. However, there is virtually no follow-up experimentation to define the mechanism. The authors discuss this, but it still remains a major weakness of this work.**

Author response: Thank you for phrasing this concern. To further advance the mechanistic understanding of the observations, we show in our revised manuscript that differences in competitive fitness of the *S*. Tm Δ*manA* mutant in mice precolonized with *E. coli* wild-type or Δ*lsrB* are directly linked to the ability of the former to occupy the AI-2 chemotaxis- dependent niche in the mouse gut (Fig 5c, lines 280-298). Furthermore, we performed mass spectrometry analysis on cecal samples from mice pre-colonized with either AI-2-chemotactic or non-AI-2-chemotactic *E. coli* strains. The concentrations of 17 monosaccharides, including mannose, were measured at days 0, 1, and 2 post-*S*. Tm infection (Fig. S7). While no significant differences were observed between the groups, the relatively modest fitness changes in *S*. Tm Δ*manA* (Fig. 5b) and the overall low abundance of mannose in cecal contents could make subtle variations difficult to detect. Consequently, we cannot rule out the possibility that niche-specific differences exist but are not captured by bulk measurements measuring average carbohydrate concentrations across diverse niches.

2. **Fig. 5: The statistics used in this experiment does not correct for Type1 errors (different time points tested); the Kruskal-Wallis ANOVA test seems more appropriate than the Mann Whitney U test used here.**

Author response: Thank you for bringing this to our attention. The Kruskal-Wallis test was performed on the data sets in Fig. 1c, 2a, 2b, 4, 5, and S1a, S1c, and S1d. The figures and figure legends were updated accordingly.

**Minor points:**

1. **Fig. 1D: these are representative images. A more rigorous, quantitative analysis would be appropriate, akin to Fig. 2B.**

Author response: The quantitative analysis of the images in Fig. 1D has now been added to Fig. S1h.

2. **Key bacterial mutants, such as the E. coli lsrB mutant and the S. Tm manA mutant, should be complemented and key assays repeated with this complemented strain.**

Author response: Thank you for this suggestion. Classical expression vector-based complementation of chemotaxis-related genes *in vivo*, especially those lacking internal promoters (like *lsrB*), is admittedly not a trivial task. However, in our previous publication, we used a co-infection model with an AI-2 overproducing *E. coli* strain (Laganenka et al, Nat Microbiol 2023). In the presence of such a strain, the luminal AI-2 concentration is high enough to abolish AI-2 gradients and, as a result, AI-2 chemotaxis. Under these conditions, the *E. coli* wild-type strain lost its fitness advantage over the Δ*lsrB* knockout, and the fitness of the Δ*lsrB* strain was restored. With this type of complementation assay, we could show the specificity of the observed *lsrB*-related phenotype.

We have now performed a similar experiment with *S*. Tm Δ*manA* vs. WT competition in the presence or absence of the AI-2 overproducing *E. coli* strain (Fig. 5c). In line with the prior experiments mentioned above, the competitive fitness of the *S*. Tm Δ*manA* mutant was higher in mice precolonized with *E. coli* Δ*lsrB*, compared to *E. coli* WT. However, the addition of the AI-2 overproducing strain restored the fitness of *S*. Tm in *E. coli* WT-precolonized mice to the level of *E. coli* Δ*lsrB*-precolonized mice. This points to the role of *E. coli* AI-2 chemotaxis in this observation and essentially represents a *manA* complementation experiment. Additionally, both *E. coli* Δ*lsrB* and *S*. Tm Δ*manA* genomes were sequenced to confirm the absence of secondary mutations (see Materials and Methods section)

**Reviewer 2**

This manuscript examines whether E. coli gut colonization, which depends on autoinducer-2 (AI-2), provides colonization resistance to the enteric pathogen Salmonella Typhimurium. The authors demonstrate that mice pre-colonized with a wildtype E. coli strain show increased resistance to Salmonella colonization, whereas this resistance is reduced when mice are pre-colonized with a ΔlsrB strain. Additionally, they test a library of Salmonella mutants for their ability to overcome AI-2-mediated colonization resistance and discover that Salmonella strains deficient in fumarate respiration or mannose utilization have reduced fitness due to E. coli AI-2-dependent niche colonization. This study presents intriguing findings.

Author response: We thank the Reviewer for the positive assessment of our work and for the helpful comments.

**Major points:**

1. **I have a concern/question that may have been addressed in previous studies, though I’m not aware of; Do the total numbers of pre-colonization matter? If there is a higher presence of E. coli in the gut, does this enhance colonization resistance against Salmonella? Have the authors tested whether the level of E. coli colonization affects colonization resistance against Salmonella. In other words, is there a certain colonization limit of E. coli (CFU count) that confers resistance against Salmonella colonization? And if so, is there a difference in CFU counts of the wildtype E. coli strain and the DlsrB mutant strain that could affect the colonization resistance against Salmonella?**

Author response: Thank you for this interesting question. In our experimental setup, streptomycin-pretreated mice are infected with *E. coli* strains 24 hours prior to the *Salmonella* challenge. Under these conditions, we would not expect the initial dose of *E. coli* to affect the outcome of the *E. coli*-*S*. Tm competition, as *E. coli* would most likely reach the carrying capacity (approximately 10^9^ CFU/g feces) within 24 hours of inoculation. We assessed this in an additional experiment in which mice were infected with 10-fold and 100-fold fewer *E. coli* CFU compared to the standard dose used throughout the study (5x10^7^ CFU). As shown in Fig. S1g, regardless of the inoculum size, *E. coli* reached the same colonization levels 24 hours after infection.

2. **Do the authors have CFU counts of the E.coli strains at days 1, 2 and 3 post infection? And how is the colonization efficiency in single infections with the E. coli wildtype strain compared to the ΔlsrB strain? If there is an initial colonization defect of the** Δ**lsrB mutant strain, does this impact the ability of S. Typhimurium to overcome colonization resistance. A previously published study (PMID:35568027) demonstrated that in co-infection experiments, a commensal E. coli and the pathogenic Salmonella Typhimurium occupy distinct microhabitats, with Salmonella-induced inflammation creating a specific niche for E. coli. This suggests that, under inflamed conditions, the CFU count of the E. coli strain may increase over time, potentially dependent on AI-2 sensing, which could, in turn, influence Salmonella’s ability to colonize the gut. Could it be that the CFU count of the ΔlsrB E. coli strain does not increase as much as that of the wildtype strain (resulting in reduced colonization resistance against Salmonella) because the mutant strain is unable to occupy its preferred habitat and cannot benefit from the nutrients made available by Salmonella-induced inflammation?**

Author response: Thank you for this interesting observation. We have now added CFU counts for both *Salmonella* and *E. coli* strains from the competition experiment, as shown in Fig. 1b (Fig. S1a-c). The changes in CFU ratios between *S*. Tm and *E. coli* WT/Δ*lsrB* are due to a slight increase in *S*. Tm CFU and a slight decrease in *E. coli* Δ*lsrB* CFU at day 4 post-*S*. Tm infection. We also assessed the capability of *E. coli* Δ*lsrB* knockout to colonize mice in single infections, and no colonization defect was observed throughout the experiment (Fig. S1d). Furthermore, no colonization defects were observed for *S*. Tm Δ*cheY* and Δ*lsrB* strains in single mouse infections (Fig. S2a)**.** We thus conclude that the observed *S*. Tm-*E. coli* competition phenotype, as shown in Fig. 1b, is likely not due to a colonization defect of the interacting strains.

**Reviewer 3**

In this manuscript, Laganenka et al. investigated the role of quorum sensing for colonization resistance to S. Tm in the mouse gut. The authors specifically analyzed colonization resistance provided by WT E. coli or E. coli deficient in AI-2 sensing. E. coli chemotaxis towards AI-2 contributed to nutrient competition, which altered the colonization resistance that E. coli provides in regards to S. Tm. AI-2 sensing deficient S. Tm. did not show reduced colonization in the presence of E. coli. Using a barcoded mutant library, authors showed that AI-2 signaling in E. coli affected central metabolism in S. Tm. A S. Tm mutant deficient in fumarate respiration showed higher fitness in the presence of WT E. coli than E. coli that cannot sense AI-2. On the other hand, a S. Tm mutant deficient in mannose utilization showed lower fitness in the presence of WT E. coli than E. coli that cannot sense AI-2.
The authors conclusions are based on rigorous study design with adequate controls and adequate samples sizes for mouse experiments. The focus of their study is a highly dynamic interplay in which they manipulated two interactors (S. Tm and E. coli), while paying attention to the response of the third (host). The authors admitted in their conclusions that a clear mechanistic understanding of the role of quorum sensing in E. coli-mediated colonization resistance to S. Tm has not yet emerged from their studies. Regardless, this study shows that quorum sensing is important in this competition between Enterobacteriaceae in the gut and provides insight into how this affects nutrient competition. It would therefore be of interest for a broad range of readers.

Author response: We thank the reviewer for recognizing the rigor in our study design and the broader implications of our findings, as well as for the helpful comments.

**Major points:**

1. The experiments are conducted to a high standard, and no additional experiments are absolutely required to increase experimental rigor. However, the manuscript would benefit from an AI-2 control experiment. Other studies previously showed that AI-2 can be introduced into the gut environment via gavage. A S. Tm + AI-2 control would allow to distinguish direct effects of AI-2 on S. Tm. metabolism (i.e. via effects on the gut environment) in the absence of E. coli compared to effects elicited by AI-2 producing E. coli.
   The authors stated themselves that a clear mechanistic understanding is still missing and requires more studies. It is unrealistic that a couple suggested experiments would provide such understanding. A revision would therefore either be a lot more experiments to achieve mechanistic understanding, or no such experiments. I opt for no more experiments and view this manuscript as a first step towards achieving such understanding.

Author response: We thank the Reviewer for their comment and agree that the manuscript should be viewed as a demonstration of the principle that quorum sensing- and chemotaxis-mediated niche occupation by one species can influence interspecies interactions *in vivo*.

We are not aware of any studies that have implemented AI-2 gavage, and in our own experiments, we did not observe an increase in gut luminal AI-2 concentrations upon oral gavage of AI-2, as measured using an AI-2 biosensor strain. However, a study by Thompson et al. 2015 (PMID: 25801025) utilized an engineered *E. coli* strain to modulate gut luminal AI-2 availability. In our previous study (Laganenka et al., Nat Microbiol, 2023), we had already implemented an AI-2 overproducing strain to demonstrate that the effect of the *lsrB* mutation *in vivo* was specifically linked to the inability of *E. coli* Δ*lsrB* to perform chemotaxis toward AI-2. We have now performed a similar experiment to link the competitive fitness of *S*. Tm Δ*manA* to AI-2 sensing by *E. coli* (Fig. 5c, lines 280-298).

To further advance the mechanistic understanding of this phenomenon, we also measured sugar availability in the cecal contents of mice pre-colonized with either *E. coli* wild-type or the Δ*lsrB* mutant (Fig. S7, lines 299-307). However, no significant differences were observed between the groups. Given that the observed changes in *S*. Tm fitness are relatively small and that differences likely exist at the niche level within the gut, such differences may not necessarily be detectable through bulk measurements.

**Minor points:**

 While no more experiments are absolutely required, the manuscript would benefit from extensive editing to increase clarity. The authors deal with a lot of different mutant combinations and different outcomes. It is at times difficult to follow the authors’ description of results. The following is a non-conclusive list of issues that require clarification.

**- Fig 2a. The description states that there is “no colonization defect” for the S. Tm lsrB mutant. This is correct, but there seems to be an increase in fitness at 4dpi in cecum samples that the authors do not discuss. Why does this mutant fare better in competition with E. coli than WT S. Tm?**

Author response: We acknowledge the apparent increase in fitness for the *S*. Tm Δ*lsrB* mutant at 4 days post-infection (d.p.i.). However, we chose not to discuss it further, as this slight increase was determined to be statistically insignificant.

**- The author’s use of “chemotaxis” and “quorum sensing” is frequently confusing, i.e. in lines 197-203. What exactly do the authors measure here?**

Author response: We apologize for not conveying the terms in a clear manner. We have now adjusted the text to provide more explanation:

“To investigate how AI-2-mediated quorum sensing (and as a result, AI-2 chemotaxis) influences the metabolic requirements for the intraluminal growth of *S*. Tm, we employed a WISH-barcoded carbohydrate utilization mutant pool. The *E. coli* non AI-2 chemotactic Δ*lsrB* mutant is impaired in AI-2 transport, thereby affecting AI-2-mediated transcriptional and post-translational regulation and chemotaxis towards AI-2. It allowed us to explore the role of AI-2 chemotaxis in the competition between *S*. Tm and *E. coli* in context of metabolic exploitation.”

**- The S. Tm cheY mutant needs to be adequately introduced. The authors expect the reader to know how this mutant responds.**

Author response: Thank you for noticing this. The *cheY* mutant is now introduced in more detail:

“Similarly to *E. coli*, *S.* Tm SL1344 benefits from chemotaxis during gut infection, as previously shown in a competitive infection model (Stecher et al., Cell Microbiol 2008). Deletion of the *cheY* gene, leading to the inability of *S.* Tm cells to follow chemical gradients, resulted in a fitness disadvantage for this strain compared to the *S.* Tm wild type. However, in contrast to *E. coli*, the fitness advantage of chemotaxis in *S.* Tm only becomes apparent with the onset of gut inflammation, and no fitness defect was observed for *S.* Tm non-chemotactic Δ*cheY* mutant in its absence (Stecher et al., Cell Microbiol 2008).”

**- Lines 132 -137. This should be described in at least a couple sentences. It does not capture that the CI differences are only observed at 4dpi.**

Author response: Corrected (Lines 139-142).

**- Line 138: “similar results” is unnecessarily vague.**

Author response: Corrected (Lines 147-148).

**- Line 141: “in line with delayed S. Tm gut colonization kinetics”. This was never mentioned previously. Experimental results should not be introduced in passing.**

Author response: Corrected (Lines 154-158).

**- Line 144: “no significant differences between the E. coli groups”. But also no difference compared to S. Tm. single infection.**

Author response: Corrected (Lines 156-158).

**- Line 156: “same strategy”. Also unnecessarily vague. AI-2 chemotaxis?**

Author response: Corrected (Line 171).

**- Line 204-214: The description lacks clarity. It is unclear how this experiment was performed, what the “seven wild-type strains” are. What is the SES and why does it need to be close to 1 to “reliably interpret the mutant fitness”?**

Author response: Thank you for this comment. We now provide more detailedinformation about the experiment:

“The *S*. Tm pool included seven wild-type strains tagged with different WISH tags to evaluate population bottlenecks during colonization, coinciding with the onset of inflammation. On days 1 and 2 post infection, the Shannon evenness score (SES) between these WISH-tagged wildtype strains was close to 1 in all mice, indicating no random loss of strains and stable *S*. Tm wild type colonization. Consequently, we could reliably interpret the mutant fitness defects in these samples. By day 3, it dropped noticeably in the case of wt *S*. Tm infections (Fig S5b). Consistent with the lipocalin-2 data shown in Fig 1c, we observed a tendency for *E. coli* to mitigate *S*. Tm-mediated inflammation, evident from the overall higher SES when *E. coli* was present (Fig S5b). The interpretation of the CIs was limited to 2 days post-infection, as a significant number of samples fell below the 0.9 SES threshold for days 3 and 4 post infection, previously established as a cutoff. Samples with SES values below 0.9 are characterized by the random loss of WISH-barcoded strains due to population bottlenecks occurring during *S.* Tm infection. This results in unreliable interpretation of mutant fitness defect data.”

**- The discussion is particularly difficult to follow. For example lines 312-332: the reference to “already mentioned above” is confusing. So what is new here? Why the two mutants, what is different between the two?**

Author response: We agree and have modified this part of the discussion section to make our points clearer:

“Finally, we identified two metabolic pathways in *S.* Tm that were affected by AI-2 signaling in *E. coli*. One of them is fumarate respiration, mediated by the fumarate reductase enzyme complex (*frd* operon). The fitness of the *S.* Tm Δ*dcuABC* mutant, lacking genes for C4-dicarboxylate antiporters and thus, like Δ*frd*, deficient in fumarate respiration, was positively affected by AI-2 signaling in *E. coli*. This was, however, only observed on day 3 of infection. During the first two days of *S.* Tm infection, we observed an opposite effect of *E. coli* AI-2 signaling on the fitness of the *S.* Tm mannose utilization-deficient Δ*manA* strain. AI-2-dependent niche occupation by *E. coli* resulted in increased pressure for *S.* Tm to metabolize mannose, resulting in lower fitness of the *S.* Tm Δ*manA* strain in mice precolonized with wild-type *E. coli* compared to those precolonized with the *E. coli* Δ*lsrB* strain. Importantly, this effect could be reversed by introducing an AI-2-overproducing *E. coli* strain into the gut lumen, which led to a saturated AI-2 response in *E. coli* wild-type cells and their inability to occupy the AI-2 chemotaxis-dependent niche. In such mice, the competitive fitness of the *S.* Tm Δ*manA* mutant phenocopied what we observed in mice colonized with non-AI-2-chemotactic *E. coli* Δ*lsrB*. These results further support the hypothesis that AI-2 chemotaxis-dependent niche colonization by *E. coli* plays a role in modulating *S.* Tm metabolism *in vivo*.”

**Reviewer 4**

In this well-written manuscript, the authors report Salmonella Typhimurium colonization dynamics change in response to E. coli sensing autoinducer-2. The manuscript provides a starting point to understand how bacteria may limit gut colonization of pathogens with similar metabolic potentials. The two major novel findings are that E. coli AI-2 sensing impacts Salmonella gut colonization dynamics and mannose utilization. The strengths of the study include the dual use of Salmonella and E. coli genetic approaches to study inter-species competition and the unbiased approach to understand the impact of E. coli AI-2 sensing on Salmonella metabolism in vivo. However, the mutants used to study AI-2 mediated chemotaxis in Salmonella are not characterized for AI-2-mediated chemotaxis, limiting the conclusions that can be made regarding Salmonella response to AI-2. In addition, there is a lack of mechanistic understanding for the role of mannose in Salmonella-E. coli gut colonization dynamics, which limits enthusiasm for the observation. Furthermore, I have some questions regarding the choices of statistical tests used, further limiting the conclusions that can be drawn from the data as presented.

Author response: We thank the Reviewer for the positive assessment of our work and for the helpful suggestions, which we have implemented in the revised version of the manuscript.

**Major points:**

1. The authors conclude that Salmonella uses chemotaxis, but not towards AI-2, for colonization of the gut using ∆cheY and ∆lsrB mutants, respectively. However, Salmonella Typhimurium ∆cheY mutants have motility defects (Bogomolnaya 2014, PLoS ONE; Rivera-Chavez 2013, PLoS Pathogens), suggesting the ∆cheY Salmonella mutant has reduced colonization due to lack of flagella-mediated motility, rather than chemotaxis itself. Furthermore, it is not clear whether AI-2 is a chemoattractant for Salmonella as it is for E. coli. Experiments to demonstrate 1. whether the ∆cheY colonization defect is due to absence of chemotaxis or absence of general motility, 2. whether or not AI-2 is a chemoattractant for Salmonella and which methyl-accepting chemotaxis proteins are responsible for AI-2 chemotaxis, and 3. testing a mutant lacking the methyl-accepting chemotaxis proteins that interact with AI-2 would all help to enhance the conclusions of the manuscript.

Author response: Thank you for this interesting suggestion. It is true that on motility agar plates, Δ*cheY* mutants appear non-motile as they fail to spread on the agar. However, this is not due to a lack of motility per se - Δ*cheY* mutant strains are motile in liquid medium (PMID: 16796690), but incapable of tumbling, causing them to become trapped in the agar. To confirm this, we performed control experiments using *S*. Tm wild type, Δ*cheY*, and Δ*lsrB* mutants grown in liquid medium and observed no significant differences in their swimming speed (Figure S2b).

Due to issues with the available batch of synthetic DPD/AI-2, we were unable to either prove or disprove the possibility of *S.* Tm performing chemotaxis towards AI-2. Although this question remains open, LsrB of *S.* Tm SL1344 still appears to be dispensable for *S.* Tm-*E. coli* competition *in vivo*.

2. **Prior work (Rivera-Chavez 2013, PLoS Pathogens) has shown that tsr and aer mediate Salmonella chemotaxis towards nitrate and tetrathionate, respectively, during intestinal inflammation. Consistent with prior work, the authors have observed a role for cheY supporting Salmonella fitness only during intestinal inflammation. Experiments to study the role of Salmonella aer and tsr in gut colonization dynamics because of E. coli AI-2 signaling would enhance the conclusions regarding the interplay between chemotaxis and inflammation.**

Author response: Thank you for this interesting observation. We agree that, in order to study the role of *S*. Tm chemotaxis in *E. coli*-*S*. Tm interactions, one could test individual MCP knockouts of *Salmonella*. However, the pleiotropic nature of such knockouts (as each MCP senses a range of chemicals, the majority of which remain unidentified) notoriously complicates data interpretation. Tsr is a good example of this, as its effect on S. Tm fitness is clearly strain-specific (PMID: 27435462; PMCID: PMC9187619; PMID: 38460128). In this case, we feel that knocking out genes for specific binding proteins (such as LsrB in the case of AI-2) represents a better strategy. However, we did not observe an effect of Δ*lsrB* deletion in *S*. Tm SL1344 in single (Fig. S2a) or competitive infections (Fig. 2a) in our model.

3. The authors demonstrate a Salmonella ∆manA mutant poorly colonizes the murine intestine, and this mutant is more fit when E. coli is present and can sense AI-2. These findings differ from published observations which show a Salmonella ∆manA mutant has no defect in colonization of the murine intestine, unless mice are fed mannose (Boulanger 2022, J. Bacteriology). Genetically linking manA with the observed colonization defect through complementation would verify the study observations. Further investigation into this phenomenon by addressing role of inflammation in Salmonella mannose utilization and establishing whether E. coli and AI-2 sensing impact gut mannose availability would provide needed context for inter-species competition with respect to mannose usage.

Author response: Thank you for noticing this interesting point. The observed discrepancies in the fitness of the *S*. Tm Δ*manA* mutant may be due to differences in the strains used in our study (*S*. Tm SL1344) compared to those in Boulanger et al. (2022, PMID: 36383008), who utilized *S*. Tm ATCC14028. Additionally, variations in the mouse models employed - C57BL/6J in our study versus Swiss Webster in theirs - could also contribute to these differences.

By saturating the chemotactic response of *E. coli* wild-type towards AI-2, achieved by introducing an AI-2-overproducing strain into the mouse gut lumen, we restored the competitive fitness of the *S*. Tm Δ*manA* mutant to levels comparable to those observed in mice pre-colonized with *E. coli* Δ*lsrB*. This demonstrates that *E. coli’s* ability to colonize the AI-2 chemotaxis-dependent niche specifically influences the pressure on *S*. Tm to utilize mannose (Fig. 5c, lines 280-298). Furthermore, sequencing confirmed the absence of non-synonymous mutations in the genomic DNA of the *S*. Tm Δ*manA* mutant.

We also measured sugar availability in the cecal contents of mice pre-colonized with either *E. coli* wild-type or the Δ*lsrB* mutant (Fig. S7, lines 299-307). No significant differences were observed between the groups. However, given that the changes in *S*. Tm Δ*manA* fitness are relatively small and that overall mannose abundance in cecal contents is low, making subtle differences difficult to detect. Differences at the niche level, which may not be captured by bulk measurements, cannot be ruled out.

**Minor points:**

It is my understanding that when there are multiple comparisons made between treatment groups within an experiment, an ANOVA or equivalent non-parametric test should be used with appropriate corrections made for multiple comparisons. In addition, analysis of the time variable should also be considered. Can the authors please explain why several Mann-Whitney U-tests were used to compare three treatment groups in figures 1C, 2A, 2B, 4, 5 and S1A, S1C, and S1D?

Author response: The Kruskal-Wallis test was performed on the data sets in Fig. 1c, 2a, 2b, 4, 5, and S1a, S1c, and S1d. The figures and figure legends were updated accordingly.
